# Social Solidarity and Herbert Spencer: Not the Oxymoron That Might Be Assumed

**DOI:** 10.3389/fsoc.2019.00001

**Published:** 2019-02-12

**Authors:** John Offer

**Affiliations:** School of Applied Social and Policy Sciences, Ulster University, Coleraine, United Kingdom

**Keywords:** Herbert Spencer, social self-consciousness, social organicism, spontaneous cooperation, Emile Durkheim, militant and industrial societies, solidarity

## Abstract

This article attempts to retrieve important aspects of Spencer's sociology from the general neglect and misrepresentation which threatens to overwhelm it all. It does touch *in passing* on many such highly dubious contentions as that he was a “social Darwinist,” but the prime focus is to deal with three linked themes. First, the article examines the significance of his attribution to individuals of “social self-consciousness” as part of sociality, thus distancing it from Durkheim's influential but suspect reading of Spencer's individuals as egoistic. Second, it rescues his concept of “the social organism” from misinterpretation. His own writings show it to be a more rigorous and suggestive attempt to configure the morphology of “the social” than commonly assumed. Third, it reconstructs the status of his contrast between “militant” and “industrial” social forms as a contrast between different but more general forms of social life that those descriptions in fact register. With the focus on these three linked themes the article improves the historical accuracy of our understanding of Spencer's sociology. It also repositions key aspects of it as not alien, quaint and a spent force, but ontologically challenging and possibly prescient for debates about the meaning of “the social” today.

## Introduction

For a long time, some critics have accused Spencer of writing things which in fact he did not write. With justice it has been said that “many find it unnecessary any longer to read Spencer's works in order to pretend to ‘know’ his social philosophy” (McCann, 2004, p. 95). So this article at the start will have to “bracket off” some stigmatizing assumptions that the name Spencer probably conjures up. One respected senior sociologist, not alone, has told us that Spencer was a “social Darwinist” and a “spent force,” a sociologist who worked with an unexamined “survival of the fittest” idea (Crow, 2005, pp. 67, 181)^1^. I will touch in passing on many such highly dubious contentions. However, the overriding aim of this discussion is to retrieve Spencer's conception of “the social.” And to get there we have to be clear about his conception of “the individual.” The first section of this article is on the “individual” and the “social” in Spencer, distancing it from Durkheim's reading of Spencer. The second section restores to view the picture of what he saw as the core of the “social organism” model, and the third rescues his “militant”/”industrial” distinction about the types of social relations from interpretative disarray. The final section suggests that this retrieval exercise has a positive role to play in current and cross-disciplinary debates about conceptualizing and researching “the social” today.

## The “Individual” and the “Social”

First, Spencer must be defended against the common claim that he was an “atomic individualist,” meaning that he denied reality to something called social life beyond regarding it as reduced to the aggregate of individual activities. Thus for R.S. Peters, Spencer's proposals on education were warped by “atomic individualism” (Peters, 1981/2015, p. 71), while earlier criticisms of Spencer (basically idealist in nature) from Durkheim highlighted what he believed to be his “narrow utilitarianism and utilitarian egoism” (given in Lukes, 1969, p. 20). For Durkheim, Spencer's thought was equated with “moral poverty”: “it is only too clear that all social life would be impossible if there did not exist interests superior to the interests of individuals,” while essentially the same claims were commonly leveled against Spencer by idealist social thinkers such as Ritchie from the late 1870s (den Otter, 1996, pp. 92–98; 151–152). However, given the large influence of Durkheim on sociology, it is on his particular associated interpretation of Spencer that this article focuses.

This part of the discussion begins with an outline of the chief relevant comments made by Durkheim on Spencer in some of his main publications. In his *The Division of Labor in Society* of 1893, Durkheim, addressing Spencer by name, argues that “we should not, as does Spencer, present social life as the mere resultant of individual natures alone, since, on the contrary, it is rather the latter that emerge from the former. Social facts are not the mere development of psychological facts, which are for the most part only the prolongation of social facts within the individual consciousness” (Durkheim, 2014/1893, p. 272). According to Durkheim, “Collective life did not arise from individual life; on the contrary, it is the latter that emerged from the former. On this condition alone can we explain how the personal individuality of social units was able to form and grow without causing society to disintegrate. Indeed, since in this case it developed from within a pre-existing social environment, it necessarily bears its stamp” (Durkheim, 2014/1893, p. 218). Durkheim enlarges on this point a few pages later, remarking that it is “doubtless a self-evident truth that there is nothing in social life that is not in the consciousness of individuals. Yet everything to be found in the latter comes from society” (Durkheim, 2014/1893, pp. 272–273). By way of completing his argument he adds, in a Note, “But because individuals form a society, new phenomena occur whose cause is association, and which, reacting upon the consciousness of individuals, for the most part shapes them. This is why, although society is nothing without individuals, each one of them is much more a product of society than he is the author” (Durkheim, 2014/1893, p. 274).

In his *The Rules of Sociological Method* (1895), Durkheim in essence maintains his position on Spencer. Durkheim declares that while Spencer sees social life as “essentially spontaneous and society is a natural thing,” when he calls social life “natural” this is simply because he finds a basis for it “in the nature of the individual” (Durkheim, 2013/1895, p. 97). While individual consciousnesses follow from the nature of the organic and psychical *being taken in isolation*, what Durkheim refers to as “collective consciousnesses” follows from the “combination of a plurality of beings of this kind.” Thus the results “cannot therefore fail to be different, since the component parts differ to this extent” (Durkheim, 2013/1895, p. 99). Indeed, with his reading of Spencer in mind, Durkheim's identification of the ontological reality of a collective consciousness takes him into judgmental territory: there is a constraint that is “normal” associated with “reflection which causes man to understand how much richer or more complex and permanent the social being is than the individual being,” revealing to him “reasons to make comprehensible the subordination which is required of him” and for the feelings of “attachment and respect which habit has implanted within him” (Durkheim, 2013/1895, p. 98).

Note also that in Durkheim's “Individualism and the Intellectuals” of 1898 he makes a marked contrast between the “egoism” of Spencer's analysis and the social nature of the analysis provided by Kant: “No one has insisted more emphatically than Kant on the supra-individual character of morality and law” (given in Lukes, 1969, p. 22, also reprinted in Hamilton, 1990). We find too the emphasis on the supra-individual character of the social reoccurring in 1917:

human societies present a new phenomenon of a special nature, which consists in the fact that certain ways of acting are imposed, or at least suggested *from outside* the individual and are added on to his own nature: such is the character of the ‘institutions' (in the broad sense of the word) which the existence of language makes possible, and of which language itself is an example. They take on substance as individuals succeed each other without this succession destroying their continuity; their presence is the distinctive characteristic of human societies, and the proper subject of sociology (given in Durkheim, 2013/1895, p. 190).

Given Durkheim's sentiments as outlined here (which are largely consonant with more general modes of idealist social thought), there is indeed room for thinking that while markets and the division of labor “clearly require a degree of trust, institutional guarantees, and regulation” in order to be stable over time, Durkheim has overstated the case “in claiming that they require an inclusive moral community” (see, for example, Lukes, 2014, p. xxvii). However, the actual criticism advanced here is in essence more specific in focus, namely that *Spencer's own thought* has been misunderstood by Durkheim. In a nutshell, Durkheim was incorrect as a matter of fact when he described Spencer's sociology as exhibiting (as Durkheim himself wrote), a “narrow utilitarianism and utilitarian egoism,” and “moral poverty,” giving as a reason for his statement that “it is only too clear that all social life would be impossible if there did not exist interests superior to the interests of individuals” (in Lukes, 1969, p. 20). The absence of a collective consciousness for Durkheim entails that there can be no norms governing contract, thus basic egoism cannot produce what is social: “a set of rules cannot function properly if it is not accepted by the individuals and … this acceptance is incompatible with a basic egoism” (Steeman, 1963, p. 65). Social life entails solidarity, which entails altruism. Altruism is the basis of social life, not an ornament to it. Thus, for Durkheim, “no real social order would be possible if the individual were really the kind of egoist Spencer makes him to be” (Steeman, 1963, p. 65). If there is a social order present, this fact goes to show that the individual cannot be purely egoistic. Thus it is impossible that the “individualizing trend in social evolution” can foster the type of society which Spencer had in mind, “a harmony of egoisms” (Steeman, 1963, p. 65).

However, the reality is that for Spencer each person was capable of possessing a “social self-consciousness” and a “chivalry” (“The morals of trade,” Spencer, 1859, pp. 140–141). His *The Study of Sociology* of 1873, a popular, influential, and a pioneering overall account of the subject, argued that as well as a rounded individual self-consciousness formed in each person in society, each person must develop a rounded impression of his or her society: “A well-balanced social self-consciousness, like a well-balanced individual self-consciousness, is the accompaniment of a high evolution” (Spencer, 1873, p. 291). Spencer saw our *self-consciousness of others* as an essential constituent of “society,” or “the social organism^2^.”

In addition, in Spencer's three-volume *Principles of Sociology*, published between 1876 and 1896, he observed that from the point of which “a combination of men acquires some permanence, there begin actions and reactions” between each member of it and the community itself, such that “either affects the nature of the other” (Spencer, 1877, pp. 12–13)^3^. Social cooperation, indeed, begins by joint defense and offense, and out of these cooperations “all kinds of cooperations have arisen” (Spencer, 1891, p. 241). For Spencer, the presence of cooperation may be understood as a sign that, in terms of psychological adaptation, ego-altruistic sentiments were emerging through gregarious life, in addition to egoistic sentiments. This was the argument in his *Principles of Psychology*^4^. Ego-altruistic sentiments themselves were influencing and in turn being influenced by the conditions within and surrounding social life, through a helical process of adaptation^5^. Spencer observes that the mere gatherings of individuals into a group does not make them into a society: a society, “in the sociological sense, is formed only when, besides juxtaposition there is cooperation. … Cooperation, then, is at once that which cannot exist without a society, and that for which a society exists. It may be a joining of many strengths to effect something which the strength of no single man can effect; or it may be an apportioning of different activities to different persons, who severally participate in the benefits of one another's activities” (Spencer, 1891, p. 244; Offer, 2015)^6^. In whatever way a society originates, mutual dependence replaces independence. In his first section of *Principles of Ethics* (with the title “The Data of Ethics,” which had originally been published separately in 1879) Spencer went on to add that (a) that conduct continues to evolve through adaptation beyond avoiding direct and indirect harm to others until “there are spontaneous efforts to further the welfare of others” (Spencer, 1910, p. i:47), that (b) human nature will be so modified by “social discipline,” that in the end “sympathetic pleasures will be spontaneously pursued to the fullest extent advantageous to each and all” (Spencer, 1910, p. i:250), and that (c) the well-being of each individual person “is involved with the well-being of all” (Spencer, 1910, p. i:216)^7^.

However, these foundational of aspects of Spencer's sociology, crucially our individual consciousnesses of others, did not register with other broadly contemporary sociologists such as Durkheim (and Tönnies, Dilthey, and Simmel) in their critical comment on Spencer.

It seems clear, though, that Spencer's “well-balanced” “social self-consciousnesses,” possessed by each person, reflecting their own circumstances and that of others around them, is other-regarding. So Spencer's position is a long way from “atomic individualism,” or what Durkheim has depicted as “narrow utilitarianism and utilitarian egoism.” It may not refer to in Durkheim's sense a collective entity, but then is that necessary?

It has been questioned whether Durkheim's theoretical premise that “a moral order—a normative infrastructure—is a necessary pre-requisite to a division of labor': all that is required “is a common or complementary set of goals among various parties, appropriate motivation, a means of communication, and the ability to coordinate efforts” (Corning, 1982, p. 315). A formula on these lines does not necessitate the presence of spiritual values and human brotherhood, components of the “solidarity” which Steeman reasonably attributes to Durkheim's position in advance of the possibility of a division of labor in social life. In practical terms it seems Durkheim makes “an ontological break between the social and the individual domain” (Meloni, 2016). To what extent Spencer himself makes an ontological break is more difficult to determine^8^. However, in fact, *pace* Durkheim, some weaker sense of sociality among individuals, which we might call civility, may be all that is required to precipitate a division of labor and interdependence. This is Spencer's position, and it is not recognized by Durkheim: “Even Durkheim's favorite utilitarian exemplar, Herbert Spencer, had argued that social equilibrium in advanced societies presupposed the adaptive acquisition of altruistic sentiments and a concern for the liberty of others” (Bowring, 2015, p. 166). Indeed, as will be commented on later, Zafirovski has noted that, since Spencer “considers that in the absence of ‘common ends,’ no cooperation and society would be possible, this implies a latent normative solution to the problem of social order” (Zafirovski, 2000, p. 556).

Durkheim's treatment of Spencer on these matters is unreliable, but it has been allowed to stifle Spencer's own voice. Durkheim eliminates without comment Spencer's own version of “moral individualism,” elevating instead the binary contrast, which Spencer had already transcended, between a community or a society as a “moral community” and as a utilitarian “network of self-interested ‘exchangists”' (Perrin, 1995, p. 352).

Although, unlike Durkheim, Spencer did not have a concept of a collective conscience, it would be a serious mistake to assume that he thus confused unplanned spontaneous order with amoral chaos. On the contrary, individuals were moral and capable of altruism. Through freedom to adapt to circumstances, individuals and societies were allowed to prosper because this capacity permitted additional flexibility, cooperation, and space for innovation in interaction (Spencer is thinking of adaptation as shaped by the presence of other individuals or societies as well as of the material or biological circumstances, Dingwall and King, 1995). To this end, Spencer had prescribed that the principal responsibility of government was to try to ensure to all equal liberty. In reality, this is the model of a society as catallaxy or spontaneous order, which Spencer probably had absorbed from the political economist Richard Whately. Whately, as I have argued elsewhere, is an underappreciated source of influence on Spencer (see Offer, 2010a). George Smith concluded in 1981 that Spencer is an important writer “in the spontaneous order school of social theory.” He added that there are similarities between Spencer and F. A. Hayek, although Hayek “pays little attention to Spencer's contributions.” In Smith's view, “Spencer's entire social theory may be seen as an elaboration of the spontaneous order model.” Spencer explicated this idea more fully than his predecessors such as “Adam Ferguson, Adam Smith, and others” (1981, p. 424).

This type of concern with the phenomena of sociality in Spencer is not open to rejection as exceptional in his thought. In fact, its centrality instead points to the views of Durkheim (and Tönnies, see Offer, 2010a, pp. 216–218; 251–252), idealist thinkers, such as see Bernard Bosanquet (on Bosanquet and Spencer see Offer, 2010a), and other writers, including J.A. Hobson and Benjamin Kidd (on both Hobson and Kidd on Spencer, see Offer, 2015), as being gravely misleading when they present Spencer as an “atomic individualist.” The “spontaneous efforts to further the welfare of others,” including the formation of voluntary co-operative associations in general which serve to express and foster fellow feeling, form, for Spencer, core aspects within the development of social life, and thus of social evolution (as the lengthy section on “beneficence” in his second volume of his *Principles of Ethics* of 1893 amply demonstrates)^9^.

## The “Social Organism”

By this point it might be objected that something crucial is being omitted. Does not Spencer's famous conception of a society as a “social organism” cut against individualism and treat individuals instrumentally as means to the holistic ends of “society”? We thus need to devote some time to how Spencer understood the idea of “society” itself. When that conception is itself examined with care a quite different understanding emerges and the apparent tension dissolves.

As well as pondering the nature of “societies” in the 1850s, Spencer was absorbed by the at once practical and conceptual problem in biology over the vexed matter of “compound individuality,” then mooted over certain organisms (discussed more extensively than here in Offer, 2015, drawing on Elwick, 2003, 2007). Spencer's essay “The social organism” (Spencer, 1860) complemented another on “The ultimate laws of physiology” of 1857. Spencer's original and preferred title for the essay was “Transcendental physiology,” so when he published later a collection of his own essays, he reverted to that title, and that is the title used here. The essay speculated on some hypothetical physiological structures beyond specimens already available within anatomy laboratories. Spencer did *not* assimilate any society or societies in general to any one known individual organism or set of organisms. He was to insist again, in his *Principles of Sociology*, that:

there exist no analogies between the body politic and a living body, save those necessitated by that mutual dependence of parts which they display in common. Though, in foregoing chapters, sundry comparisons of social structures and functions to structures and functions in the human body, have been made, they have been made only because structures and functions in the human body furnish familiar illustrations of structures in general. The social organism, discrete instead of concrete, asymmetrical instead of symmetrical, sensitive in all its units instead of having a single sensitive centre, is not comparable to any particular type of individual organism, animal or vegetal (Spencer, 1876, p. 613).

The main subject of “Transcendental physiology” was on the nature of *forms* of organisms and their physiologies, and their modification by the forces associated with the exercise of functions. Thus the essay explored, said Spencer, “sundry laws of development and function which hold not of particular kinds or classes of organisms, but of all organisms: laws, some of which have not, we believe, been hitherto enunciated” (Spencer, 1857, p. 63).

Spencer's 1857 essay itself made morphological comparisons between social phenomena and physiological phenomena: there were general principles of development and structure found in organized bodies and in societies. A match between “the broad facts of both physiology and sociology” emerged “not as a plausible fancy, but as a scientific truth” (1857, pp. 101–102). In Spencer's “new” physiology, physiology and sociology will “more or less interpret each other.” The study of how cause and effect relations actually exist in “the social organism” may inspire the search for kindred examples in the individual organism, yielding explanations hitherto unavailable. Similarly, the physiologists' understanding of growth and function may give “the clue to certain social modifications otherwise difficult to understand.” In Spencer's opinion, the subject areas in question will “exchange suggestions and confirmations; and this will be no small aid” (1857, p. 102). No “supernatural” source was any longer required to furnish a foundation for understanding “society,” “life,” or “mind,” and thus a subject of sociology was possible.

However, the upshot was that one real and scene-shifting peculiarity possessed by the “social organism” emerged: “while in the body of an animal only a special tissue is endowed with feeling, in a society all the members are endowed with feeling” (1860, p. 276). The contrast pinpointed constitutes one of absolutely fundamental importance to Spencer's model of society as organism: in individual bodies “the welfare of all other parts is rightly subservient to the welfare of the nervous system.” But this does *not* apply to societies. Because of the fact of individual consciousnesses, “Corporate life in bodies-politic,” says Spencer, “must therefore serve the lives of the parts” (Spencer, 1860, pp. 276–277). Scarcely less important was that he recognized that the members were mobile, or “locomotive.” Spencer was not by name mentioned in a recent theoretical study by Elder-Vass into the causal powers of social structures. However, this particular fact of mobility as a structural feature which, as it happened, Spencer had identified, has surfaced again to figure with some prominence in this new study: at least some varieties of social structures “do not depend on spatially specific physical relations between their parts to produce the mechanisms that give them their causal powers.” They can be “spatially disarticulated—they can operate in the absence of any specific set of spatial relations between their parts” (2012, p. 200). Spencer understood this, and it seems that he may have seen that this absence of a specific spatial relation facilitated the membership of more than one social group, the occurrence of intersectionality, as a consequence.

By 1860, then, the ferment of intellectual preparation which lay behind Spencer's particular conception of the “social organism” had furnished him with a more robust and interlocking set of arguments than his reputation in some quarters as a dilettante gives him credit. A society was stipulated to be an organism, yet with singular characteristics. Societies, then, are “organisms” and thereby a constituent of the natural world, but essentially *sui generis* in the nature and powers of its units, structures and functions (they are therefore “super-organisms”). For Spencer, that was sufficient to underpin his fundamental aim of establishing a scientific sociology (Spencer, 1896, p. 315; Offer, 2015).

It has often been said that conceptions of societies as organisms have accompanied attempts to encourage reformist or conservative social and political arrangements, in each case with requests for additional intervention by government. There are, though, no necessary or “natural” connections here: parallels between organisms and societies could be legitimately formulated to point to no role for government save a “negatively regulative” role. That is to say, that *government* should not interfere with the exercise of someone's liberty unless that exercise would infringe the liberty of another to do likewise. To an article written by T. H. Huxley Spencer replied (citing Whately again):

Far from contending for a laissez faire policy in the sense which the phrase commonly suggests, I have contended for a more active control of the kind distinguishable as negatively regulative. One of the reasons I have urged for excluding State-action from other spheres, is, that it may become more efficient within its proper sphere (Spencer, 1871, p. 438).

This “negatively regulative” formulation is the one which Spencer in fact adopts, reminding us that since social individuals have social self-consciousnesses there is no social sensorium in society. Given the richness of Spencer's conception of individuals, he can fuse it with an organicist (note, not mechanistic) theory of social life, but with government occupying a limited “proper sphere”: “the individual is not to be seen as isolated or separate, but is rather socially situated, recognizable only through his connections to others in the society. Spencer's man is not Hobbes's man, nor is Spencer's individualism Hobbes's individualism, for Spencer is resolute in his rejection of the atomic postulate” (McCann, 2004, pp. 125–126). Both the logical coherence of Spencer's position and the misreading of it by many of the criticisms made of it, have been succinctly put by Gray (Gray, 1996, p. 233):

At the root of the critics' misinterpretation of Spencer's theory was their mistaken assumptions that the ‘opposite’ of individualism was organicism, and that the ‘opposite’ of ‘organicism’ was individualism. The truth is, however, that if there is an ‘opposite’ of individualism it is collectivism, not organicism, and if there is an ‘opposite’ of organicism, it is mechanism not individualism. Spencer consistently developed an individualistic/organicist theory, and consistently opposed it to a collectivist/mechanistic theory.

The idea of the social organism in Spencer's hands was in principle a stimulating integration into a single model of the ideas of spontaneous cooperation and relatively permanent but mutable social structures as the bedrock for the interpretation of society and social life^10^. There can be little room for doubt now that it was indebted to his cross-disciplinary reflection on the state-of-the-art controversies of biological science of the 1850s arising from the apparently prolific diversity of physiological phenomena and organic forms. Once it is accepted that it needs to be interpreted in the context of his “transcendental physiology” Spencer's “social organism” is revealed, as he intended, as a means to unifying the study of forms in all that had lived, and a worthwhile contribution to sociology.

## “Militancy” and “Industrialism”

Spencer's categorization of social relations into two differing types, “militant” and “industrial,” played an important part in his sociology. The contrast provided a focus to the comparisons and dissimilarities exhibited between various societies, while entertaining the belief that civilized societies in the future would resolve their differences, making for a peaceful political order internationally. While its essence is present in 1842 in the Letters collected together in *The Proper Sphere of Government*, Spencer's first substantial publication (Spencer, 1843, p. 23), the first formally developed statement of the contrast came in *First Principles* of 1862, when he discussed social metamorphoses among “civilized nations,” in particular on changes “from the military or predatory type of social structure, to the industrial or mercantile type” (Spencer, 1862, p. 190, note *social structure*, not society)^11^. As the old lines of organization dissolve, new ones surface. In the first volume of the *Principles of Sociology*, Spencer clarifies these militant and industrial forms in their “pure” forms by highlighting the juxtaposed versions of co-operation they present. He says that the co-operation “by which the life of the militant society is maintained, is a compulsory co-operation” (Spencer, 1877, p. 584, note that reference is now to society). The industrial type of society displays a voluntary co-operation in “its multiform activities” (Spencer, 1877, pp. 589–590). Spencer qualifies how the meanings of “militant” and “industrial” are to be understood in the second volume of the *Sociology*. First, it would be false to assume that the industrially-organized society (Spencer, 1891, p. 604).

is one in which, of necessity, much work is done. Where the society is small, and its habitat so favorable that life may be comfortably maintained with but little exertion, the social relations which characterize the industrial type may co-exist with very moderate productive activities. It is not the diligence of its members which constitutes the society an industrial one in the sense here intended, but the form of cooperation under which their labors, small or great in amount, are carried on.

In fact, Spencer's message might have been better presented and understood with less emphasis on “militant” and “industrial” in the distinction, and more on the two contrasting forms of cooperation in social relations themselves. The description and analysis of social relations in terms of differing forms of cooperations, whether in domestic, ceremonial, religious, professional and moral contexts, as well as in the contexts of political priorities and structures, were his central focus. Seen in that light, Spencer's contribution has more in common with the orientation of the later distinction between “Gemeinschaft” and “Gesellschaft” social relations proposed by Tönnies (1887) than is usually recognized, and possibly also compared with Durkheim's distinction between “mechanical” and “organic” solidarity in *The Division of Labor in Society*.

Spencer's emphasis on the differences between the *forms* of social relations themselves in making his militant/industrial contrast reinforces his second important qualification about the interpretation of the concepts “militant” and “industrial.” He distinguishes the industrial type of society, “properly so called,” from the type “in which the component individuals, while exclusively occupied in production and distribution, are under a regulation such as that advocated by socialists and communists.” For this also involves “the principle of compulsory cooperation,” since individuals are “prevented from competing with one another in supplying goods for money” (Spencer, 1891, pp. 604–605).

A little more detail is needed on the chief characteristics of the two types of social relations. In militant social relations, where the purposes of offense and defense are pre-eminent, the forces of individuals have to be combined to act in concert, whether directly or indirectly. A process of regimentation according to status takes place in the army. Social relations in the community at large are shaped to serve the needs of the army. The result is that “the individual is owned by the State. While preservation of the society is the primary end, preservation of each member is a secondary end—an end cared for chiefly as subserving the primary end” (1891, p. 572). Under militancy, citizens become used to obedience and unused to the exercise of the powers of initiation (1891, p. 602). Actions as such are associated with obedience to the personal orders of figures with authority. In cultural terms, this course of life, “which makes personal causation familiar and negatives experience of impersonal causation, produces an inability to conceive of any social processes as carried out under self-regulating arrangements” (1891, p. 602).

However, where the corporate action of a militant regime ceases to be needed to protect the corporate body from the destruction of its member individuals, industrially organized social relations are paramount. The individual, “instead of being sacrificed by the society, has to be defended by the society” (1891, p. 607). Spencer means that the end which remains for corporate action is that of “preserving the component members of the society from destruction or injury by one another: injury, as here interpreted, including not only immediate, but also remote, breaches of equity” (1891, pp. 656–57). This function of corporate agency is that of administering “justice” (1891, p. 638). Processes in “industrial” societies are characterized as having spontaneously generated social relations which possess loose central controls, and in which socially self-conscience private individuals are able to pursue “decentered” ends, rather than having a central control directing those interchanges between private individuals toward collective normative purposes (see Dingwall and King, 1995).

There is serious divergence in the commentary on Spencer's militant/industrial contrast. Whilst it is said that its importance for Spencer's sociology is “hard to overestimate” (Bell and Sylvest, 2006, p. 225), there is at first glance rather slender evidence for a specific claim that the militant/industrial distinction is a key part of Spencer's *directional theory of the evolution of societies*, although this is often made. There seem to be only three occasions on which, on the face of it, Spencer might be in this position.

In the *Principles of Society*, he referred to “the communal proprietorship of land.” This, Spencer says, was “partially or wholly merged in the ownership of dominant men during evolution of the militant type,” and he then adds that communal proprietorship “will be resumed *as the industrial type becomes fully evolved”* **(**1891, p. 556 emphasis added)^12^.

In the same book, again with the rather distant future in mind, he suggested that, just as the difference between militancy and industrialism is shown by the substitution of the belief that individuals exist for the benefit of the State for the belief that the State exists for the benefit of individuals; so the contrast between the industrial type and *the type likely to be evolved from it*, is shown by the substitution of the belief that life is for work for the belief that work is for life (1877, pp. 595–596)^13^.

Finally, in *The Man vs. The State*, Spencer claimed that at every stage of social evolution “there must exist substantial agreement between practices and beliefs—real beliefs I mean, not nominal ones” (Spencer, 1884, p. 170). Thus the pathway to the fully developed industrial type had to involve experience of the discipline and the customary hierarchical order which was generated through the application “in long ages” under “the militant type,” and also “the willingness to act under direction (now no longer coercive but agreed to under contract)” (Spencer, 1884, p. 172)^14^.

Notice, though, that on these particular occasions Spencer is indeed referring to changes generated “in long ages.” Naturally, in the long run it was the case that nothing was exempted from evolutionary change in Spencer's theoretical perspective. In that specific sense the militant/industrial contrast itself could cease to possess salience in a world that had transcended it. I will return to this point later.

However, when he ordinarily used the contrast it was in the context of a *relatively* compressed time scale (say the last 2000 years or so). The fundamental use of the militant/industrial contrast was to simply isolate and capture patterns of similarity and difference over how cooperation in particular occurred in the social life, within and between societies.

Hence in the *Principles of Sociology*, Spencer announced that societies may be grouped into “the predominantly militant and the predominantly industrial—those in which the organization for offense and defense is most largely developed, and those in which the sustaining organization is most largely developed” (Spencer, 1877, p. 570). This division flags up the kinds of social activity which have preponderance at a particular time and place, and the resulting contrasts in the organization possessed by the society in question. When coercion or compulsion is behind cooperation, by class or *status*, contrasts with voluntary cooperation, and *contract* (Spencer uses these concepts, attributing them rightly to Sir Henry Maine, Spencer, 1896, p. 484)^15^.

Again, in *The Study of Sociology*, he referred to the *changes* in the structure of a society specifically associated with any alteration “in the ratio of the predatory to the industrial activities” (1873, p. 347). Moreover, when Spencer composed his mature statement of the militant/industrial contrast, in the “Political Institutions” section of the *Principles of Sociology*, he says that, during “social evolution,” “there has habitually been a mingling of the two” (1891, p. 568).

Note too that traffic between the two is not one way. There are transformations “of the militant into the industrial and the industrial into the militant.” As it happens, since Spencer's own sympathies were unequivocally with the voluntary cooperation of the industrial type, which was under pressure, he felt, by the 1870s, he wrote mostly on how the industrial type “retrogrades toward the militant type if international conflicts recur” (Spencer, 1877, p. 600).

So how best should the militant/industrial contrast be interpreted? Wiltshire insists that Spencer's scheme of social evolution possessed definite stages, with progress “from the ‘militant' to the ‘industrial’ type of society.” Spencer, claims Wiltshire, *rejected* the possibility that a society could exhibit characteristics of both types at the same time for the reason that the “‘industrial’ type is the ‘perfect’ state”' (Wiltshire, 1978, p. 247). However, Spencer's own words, as we have seen, convey the opposite message, with the two types “mingling.” The entry in the *Oxford Dictionary of National Biography* on Spencer by Jose Harris is ambushed on the same grounds: the premise of his social theory was that “industrialization and militarism were mutually exclusive” (she adds that, with absurdly misplaced condescension, “the most cursory glance at the history of nineteenth-century Europe and America might have suggested otherwise).”

Weinstein has interpreted Spencer's types in the context of four phases of sequential “development” in political history: “(1) primitive societies characterized by informal political cooperation, (2) ‘militant’ societies dominated by regimented, centralized political authority, (3) ‘industrial' societies distinguished by the relaxation of centralized political authority and the emergence of liberal values and, finally, (4) liberal utopias where government withers away” (Weinstein, 1998, p. 16). Spencer expresses hopes for a future liberal utopia, but otherwise Weinstein's “phases” move well beyond Spencer's own words. Wiltshire, Harris and Weinstein have regrettably overlooked that Spencer was clear that “advanced societies” of his time were of a “semi-militant semi-industrial type” (Spencer, 1884, p. 170; Offer, 1994)^16^.

Turner has a different reading of Spencer on the militant and industrial types, declaring that the distinction is not an evolutionary classification, as has been argued. It is, rather, “a way to fine-tune the description of a society, whatever its stage of evolutionary development” (Turner, 1985, p. 93). Spencer, Turner observes, recognized that political centralization “is a fact of social life in all types of systems, from very simple to complex ones” and that “industrial” denotes “the degree to which operative and distributive processes are free from extensive political regulation” (Turner, 1985, p. 79).

This interpretation does aid our understanding of how Spencer applied the concepts to depict the kind of balances inherent in how social life is actually conducted in a particular society and at a particular historical moment. Turner, though, over-simplifies the point, as I will try to explain. There is a definite change of mood between the first volume of the *Sociology* of 1876, when Spencer makes a bold directional claim for his theory of social evolution, referring to “that state of permanent peace to which civilization is tending” (Spencer, 1893, p. 588), and the third volume of 1896 wherein caution is in the ascendancy, as he warns that “social progress is not linear but divergent and re-divergent” (Spencer, 1896, p. 325). However, the *ultimate* evolutionary trend toward the elimination of militancy is not withdrawn. This observation and the other observations on Spencer's directional theory of the evolution of societies and militancy raised earlier in this section leave a problem for Turner.

Moreover, according to Spencer there is also another angle from which the militant/industrial division becomes infused with evolutionary dimensions, given his theory. It should be noted that when explaining in *First Principles* the part of his general theory of evolution which Spencer calls the “rhythm of motion,” he refers to the nature of antagonism between one society and another: this antagonism results “not in an uniform motion, but in an intermittent one. War, exhaustion, recoil—peace, prosperity, and renewed aggression:—see here the alternation more or less discernible in the military activities of both savage and civilized nations. And irregular as is this rhythm, it is not more so than the different sizes of the societies, and the extremely involved causes of variation in their strengths, would lead us to anticipate” (Spencer, 1862, p. 330)^17^. Thus this additional aspect to how Spencer's theory of social evolution is actually applied to social life is a further problem for Turner's reading of Spencer.

As we have already stated, Spencer's position is that once “a combination of men acquires some permanence, there begin actions and reactions” between the community and each member of it, such that “either affects the nature of the other” (Spencer, 1877, pp. 12–13). Thus the phenomena which the concepts “militant” and “industrial” signify cannot but be bound up with the actions and reactions with the community, other societies and nature in general to which Spencer had referred. But there was nothing that implied that Spencer had reified “the militant” and “the industrial” themselves into direct and linearly operative “agents” in his theory of evolution. As Turner says, the militant/industrial classification is *not* an “evolutionary classification” in the sense of being an integral component of Spencer's *theory* of evolution as such. It was also, as Turner says, a way “to fine-tune” descriptions of societies, or of parts of them. However, what Turner's reading does *not* bring out, but is true for Spencer, is, first, that what the ideas of “militant social relations” and “industrial social relations” will substantively cover will in fact change during the processes of adaptation to surrounding conditions associated with social evolution (taken to include the effects of the “rhythm of motion”), and second, that social evolution itself as a process, as conceptualized by Spencer, could make the whole idea of militant social relations redundant in due course^18^.

## Conclusion

By looking afresh at what Spencer himself said about “individuals” in (a) the context of their possession of a “social self-consciousness,” (b) their uniquely privileged status in Spencer's own bespoke concept of the “social organism,” and (c) in industrial (and militant) social relations, this article has tried to shed new light on key aspects of his sociology and retrieve a sense of its relevance to some contemporary theoretical issues. As mentioned at the start, this article has not been intended to address questions such as the precise nature of his views on the mechanisms and direction of “evolution” and the inappropriateness of labeling him a “social Darwinist^19^.”

Sociology bulks large in Spencer's output, but he was also contributing to biology and very significantly to psychology in particular, extending its horizons in the direction of a more developmental associationism (this work on both subjects is mostly neglected by sociologists, but according to the topics reviewed here, the relevant aspects were indicated). And although in the strict logic of the topic coverage of his ten-volume *System of Synthetic Philosophy* sociology was not “special” to Spencer, in practice it was outstandingly special, no less so than for Durkheim.

The distinguished sociologist Alvin Gouldner once reminded us of the value of knowing the true history of our subject: “A science ignorant of its founders does not know how far it has traveled nor in what direction” (Gouldner, 1973, p. 370). So what does this exercise in catching up tell us?

First a few cautionary remarks are needed. We have seen that although there is no firm evidence that Hayek was influenced by Spencer, it has quite often been observed, especially of the areas of Spencer's thought discussed in this article, that there is a palpable affinity (Smith, 1981; McCann, 2004)^20^. However, ideas such as “social self-consciousness” need not be confined to serving an explicitly political “neo-liberal” approach to sociology. In this respect, there are also affinities with rational action theories such as that of Coleman (1990), but Spencer differs in stressing a social normativity which has been developing through time in an increasingly “thick” way. There is too a selectionist approach in sociology, applying and adapting the Darwinian theory of natural selection to the task of explaining actual outcomes in social life. The fact that a person, government or culture *intends* something to occur does not trump the selective process: the cause alone cannot determine the outcome, the outcome has to “fit” with the selective environment (see Runciman, 1997 and Offer, 2010b on the theory, Room, 2011; Vollmer, 2013 for applications). However, the substantial differences in theory between Darwin and Spencer seem to pose at least for now an obstacle to any revival of interest in Spencer in that selectionist approach.

More promising for the present is to see Spencer not as a neglected member of a recent theoretical “school,” but as a resource through which we look afresh at our understanding of the ontological orientations of “the social” available to sociology.

With this point in mind, when the idea of the social organism was discussed reference was made to Elder-Vass's study *The Causal Powers of Social Structure: Emergence, Structure and Agency*. That reference made a real but perhaps unexpected connection between an important contribution to new perspectives on the concept of structure on the one hand and Spencer's recognition that individuals were mobile within “the social organism” on the other. However, the idea of a dormant but potentially fruitful link could be developed further. For Spencer himself illustrated what Elder-Vass has recently described as the social power of a “normative circle” in thinking about social structure. The members of such a circle form a structural entity at work in an area of social life which causes, in part, an outcome which the commitment to endorse and enforce practices with each other “makes a norm circle more effective than the sum of its members would be if they were not part of it” (Elder-Vass, 2010, p. 123). Given that one of Spencer's concerns is with how governments, and everyday habits and fashions, could serve to channel the exercise of liberty in a myopic manner, he gives us a period gem of an instance in his *Principles of Psychology*: “Consider how difficult it would be to get a lady to wheel a costermonger's barrow down Regent-street, and how easily she may be led to say a malicious thing about some lady she is jealous of—contrast the intense repugnance to the one act, which is not in itself reprehensible, with the feeble repugnance to the other act, which is itself reprehensible; and then infer how great is the evolution of the moral sentiments yet required to bring human nature into complete fitness for the social state” (Spencer, 1881, p. ii:606). In this case we may say that the normative circle has a view of “the normally expected attitudes and behavior of ladies who are members of a certain social standing.”

Elder-Vass's study also distinguished *emergent* properties from *resultant* properties, again relevant to the case of Spencer. There is general agreement that Durkheim thought in emergentist terms about “the social,” and its ability to be a causal force, in the sense that what is called “the social” has properties over and above the parts composing it (the component individuals). The contrast with resultant properties points to properties of a whole “that *are* possessed by its parts in isolation, or in an unstructured aggregation” (Elder-Vass, 2010, p. 17). Now as it happens, when this contrast has been deployed in sociological analysis, Spencer has tended to be labeled a resultant theorist insofar as “the social” is concerned. In Ingold's opinion, for example, “Spencer's society was a resultant, not an emergent, containing nothing that was not already prefigured in the properties of its original constituents” (Ingold, 1986, p. 224). His verdict though does not take into account the possession and application in social life of “social self-consciousness” by Spencer's individuals, which was discussed earlier in this article. That social self-consciousness itself was and is engendered by social life. The nature of it will vary according to the perceptions held by the members of the society in question of the relevant strength and weakness of what Spencer called “militancy” and “industrialism” in the social relations of the society. We thus seem to be faced with social life for Spencer as an emergent rather than a resultant property. This would matter even if Spencer was to be seen simply of historical interest in sociology. But it is of larger and enduring significance: it goes some way to stymie attempts to disregard Spencer as a serious sociological theorist, and one who still has original and stimulating ideas on sociality, solidarity, individualism and social structure to share.

If we take a broader perspective, we ought to remember that, with Spencer assuming no more than “common ends” and “shared conventions” as underlying cooperation in individual behavior, in contrast with Durkheim, it can be argued that we come to a point of convergence which meets with “Durkheim, Parsons, and other holists emphasizing the role of ‘normative consensus”’ (Zafirovski, 2000, p. 560). Normative convergence:

between the sociological individualism of Spencer–Hayek–Mises and Durkheim–Parsons' holistic sociology only confounds the paradox of the latter's claim of independence and irrelevance of Spencer for his ‘action theory'.…In any event, all these otherwise divergent theorists have been shown to converge, albeit in varying degrees, upon a certain point: the status of social norms, values and institutions (Zafirovski, 2000, p. 573).

Indeed it should be noted that in “Two ontological orientations in sociology,” Karakayali (2015) has assessed the place of Spencer not only in the context of that article's title, but with a discussion also of his associated commitment to locating “the social” alongside the organic sphere. That aspect itself, in a wider kind of way, is attracting attention, with a pragmatic and porous rather than inflexible ontological relationship being advocated between the “biological” and the “social,” leaving behind perhaps the nature/nurture dichotomy (Meloni, 2014).

Spencer did not as a matter of principle ring-fence sociology from psychology and biology. This article has discussed his distinctive, stimulating and challenging understanding of how best to interpret “the social” and “society.” His breadth of openness toward the sources which yielded his novel insights into these and associated concepts may well strike us now, not as alien and quaint or as a “spent force,” but as surprisingly prescient. In short, at this level of theoretical work, Spencer, properly understood, should still prove instructive.

## Author Contributions

The author confirms being the sole contributor of this work and has approved it for publication.

### Conflict of Interest Statement

The author declares that the research was conducted in the absence of any commercial or financial relationships that could be construed as a potential conflict of interest.

## Footnotes

^1^The book by the polemical historian of ideas Richard Hofstadter, Social Darwinism in American Thought (1944) did damage to Spencer's name by describing his thought as “social Darwinism.” On the different and serious levels of misrepresentation involved see Hodgson (2006), Taylor (2007, pp. 148–150), Leonard (2009), and Offer (2014). On the broader critical reception of Spencer's work, see Offer (2000).

^2^It is doubtful whether Spencer assumes that all individuals would have *identical* impressions of the community in question.

^3^The volumes of the *Principles of Sociology* form part of a series of books that developed Spencer's general theory of progressive “evolution”: *First Principles* (1862), *Principles of Biology* (2 vols, 1864 and 1867), *Principles of Psychology* (1872), *Principles of Sociology* (3 vols, 1876, 1882, and 1896, and *Principles of Ethics* (2 vols, 1892 and 1893). There were multiple editions of the titles. There are some changes of substance between editions of *First Principles* and the first volume of the *Sociology*, explained in the text.

^4^First published in 1855, then revised in 1872. Its influence on psychologists including Douglas Spalding and Hughlings Jackson is discussed in Young (1970), Rylance (2000), and Offer (2010a).

^5^Spencer's mechanism of social change described in this paragraph is derived from the Lamarckian inheritance of acquired characteristics. The focus on actions and reactions between the community and the members by Spencer might be counted as a forerunner of the problems associated with “central conflation” of such ideas as agency and structure, and agency and culture, identified by Archer (1988, see too Elder-Vass, 2010). Attempts to merge the two in Spencer's manner appear to mean it becomes logically impossible in reality to specify particular occasions when it is “more” community or “more” members taking the lead in change. However, he was attempting to refer to differing actions and reactions *through time*. Note too that Spencer denied free-will in the *Principles of Psychology*, in all editions of the book (Offer, 2010a, pp. 166–170).

^6^Durkheim quoted the first sentence given here in the *Division of Labor* (2014, p. 217). He restates that “society” and “association” must precede “cooperation” (Durkheim enlarges on his understanding of “association” in *The Rules of Sociological Method* (2013, p. 86).

^7^As noted the *Principles of Ethics* was first published in complete form in the 1890s (by Williams and Norgate). The 1910 edition was issued by Appleton in New York and London. This survey of Spencer's work is not centrally concerned with his *social or moral theory* as opposed to his sociological theory. Like John Stuart Mill in his *On Liberty* (1859), Spencer tended to worry about the threat to social life and freedom posed by the “tyranny of the majority” rather problems that might be confronting “social solidarity” as such, or “social abnormality,” as was the case with Durkheim. As has been established, however, he had his own distinctive account of cooperation, ego-altruism, and morality in social life. It may be that publications such as *Altruism, Morality, and Social Solidarity*, edited by Jeffries (2014), mark a new age of interest in the general area, and certainly Spencer should be embraced by it.

^8^Unlike Durkheim, in his own work Spencer makes no references to a “collective consciousness” or a “general will.” However, he does clearly attribute causal properties to social structures, while seeing them as mutable (as discussed in the next section).

^9^Hiskes (1983) traces the treatment of Spencer's idea of social individuals and his liberal idea of community since his day, criticizing some libertarian sources, including Robert Nozich's *Anarchy, State, and Utopia* (1974), for ignoring the altruistic attitudes and motivations of people which Spencer had described in detail.

^10^When compared with other organisms, the structures and functions “of the social organism are obviously far less specific, far more modifiable, far more dependent on conditions that are variable and never twice alike” (Spencer, 1873, p. 58).

^11^The passage is unchanged in the sixth and final edition of 1900, except by then that “or predatory” and “or mercantile” are omitted.

^12^In *Social Statics*, dated 1851 but published late in 1850 according to Spencer, Spencer had supported land nationalization, but now he regarded this support as premature, given that industrial social relations were not yet firmly established. The American social critic, journalist, and political economist Henry George, angered by the change, publicly criticized Spencer for backsliding in 1892 in *Herbert Spencer: A Perplexed Philosopher* (see Paul, 1982).

^13^These claims survive in the 1893 edition of the first volume, at p. 563.

^14^In similar vein, in the third volume of the *Sociology* of 1896 Spencer writes that, as fast as society advances, “and especially as fast as the militant type yields place to the industrial type, a centralized and coercive control, political and ecclesiastical, becomes less needful, and plays a continually decreasing part in social evolution; yet the evidence compels us to admit that at first it was indispensable” (Spencer, 1896, p. 310).

^15^In this general context, though, it is perhaps clearer to refer to “types of social relations” rather than “types of society.”

^16^A description Spencer repeated in 1896 (Spencer, 1896, p. 542), made in connection with his conditional and favorable observation that trade unions can prevent employers doing unjust things to wages paid.

^17^In the 1867 edition this passage is at p. 267. On this point Spencer was open to the charge that his law of evolution risked becoming an empty cliché.

^18^Thus while I agree with the basic claim by Dingwall and King (1995) that there is ambiguity in Spencer as to whether he regards the relationship between these two ideal types of “militant” and “industrial” “societies as evolutionary,” I here add substantiating grounds for the claim.

^19^Not least because Spencer is a pre-Darwinian thinker in the sense that he presumes design is the world in his theory of general and progressive evolution, whereas Darwin's theory of natural selection (variations combined with a struggle for existence) in the *Origin of Species* did not. In the *Principles of Biology* of 1864 Spencer presented Darwin's natural selection as the “survival of the fittest,” although the two expressions were *not* given identical meanings. Spencer continued to believe in the Lamarckian inheritance of acquired characteristics as a mechanism of change, though he also used from time to time thereafter the “survival of the fittest” (see Offer, 2014).

^20^Gane deals with Hayek's criticisms of Comte and Durkheim, but does not comment on how often Hayek, unawares, is echoing Spencer on spontaneous cooperation, and on altruism, which is not to be identified with “solidarity” in the sense of a holistic unitedness associated with Durkheim (Gane, 2014, p. 1101).
